# Tsg101 UEV Interaction with Nedd4 HECT Relieves E3 Ligase Auto-Inhibition, Promoting HIV-1 Assembly and CA-SP1 Maturation Cleavage

**DOI:** 10.3390/v16101566

**Published:** 2024-10-02

**Authors:** Susan M. Watanabe, David A. Nyenhuis, Mahfuz Khan, Lorna S. Ehrlich, Irene Ischenko, Michael D. Powell, Nico Tjandra, Carol A. Carter

**Affiliations:** 1 Department of Microbiology & Immunology, Renaissance School of Medicine, Stony Brook University, Stony Brook, NY 11794, USA; susan.watanabe@stonybrook.edu (S.M.W.); lorna.ehrlich@stonybrook.edu (L.S.E.); iischenko@hotmail.com (I.I.); 2 Biochemistry and Biophysics Center, National Heart, Lung, and Blood Institute, National Institutes of Health, Bethesda, MD 20892, USA; david.nyenhuis@nih.gov (D.A.N.); tjandran@nhlbi.nih.gov (N.T.); 3 Department of Microbiology and Immunology, Morehouse School of Medicine, Atlanta, GA 30310, USA; mkhan@msm.edu (M.K.); mpowell@msm.edu (M.D.P.)

**Keywords:** ESCRT, HIV-1, Nedd4, Tsg101, UEV domain, ubiquitin, HECT domain, E3 ligase

## Abstract

Tsg101, a component of the endosomal sorting complex required for transport (ESCRT), is responsible for recognition of events requiring the machinery, as signaled by cargo tagging with ubiquitin (Ub), and for recruitment of downstream acting subunits to the site. Although much is known about the latter function, little is known about its role in the earlier event. The N-terminal domain of Tsg101 is a structural homologue of Ub conjugases (E2 enzymes) and the protein associates with Ub ligases (E3 enzymes) that regulate several cellular processes including virus budding. A pocket in the domain recognizes a motif, PT/SAP, that permits its recruitment. PT/SAP disruption makes budding dependent on Nedd4L E3 ligases. Using HIV-1 encoding a PT/SAP mutation that makes budding Nedd4L-dependent, we identified as critical for rescue the residues in the catalytic (HECT) domain of the E3 enzyme that lie in proximity to sites in Tsg101 that bind Ub non-covalently. Mutation of these residues impaired rescue by Nedd4L but the same mutations had no apparent effect in the context of a Nedd4 isomer, Nedd4-2s, whose N-terminal (C2) domain is naturally truncated, precluding C2-HECT auto-inhibition. Surprisingly, like small molecules that disrupt Tsg101 Ub-binding, small molecules that interfered with Nedd4 substrate recognition arrested budding at an early stage, supporting the conclusion that Tsg101–Ub–Nedd4 interaction promotes enzyme activation and regulates Nedd4 signaling for viral egress. Tsg101 regulation of E3 ligases may underlie its broad ability to function as an effector in various cellular activities, including viral particle assembly and budding.

## 1. Introduction

The product of the tumor susceptibility gene 101 (Tsg101) is a broadly conserved and early-acting factor in cellular ESCRT (endosomal sorting complex required for transport) machinery [Tsg101: reviewed in [1,2,3]; ESCRT machinery: reviewed in [4,5,6]]. Its N-terminal domain resembles ubiquitin (Ub)-conjugating (E2) enzymes except that Tyr replaces Cys in the active site, rendering it unable to catalyze the transfer of Ub to Ub-ligating (E3) enzymes. The protein is thus designated as a Ub E2 variant (UEV) [7]. The UEV domain also houses a pocket that recognizes a short proline-containing motif, Pro-Thr/Ser-Ala-Pro (PT/SAP), found in both cellular and viral-encoded proteins (designated as the “late domain” in the latter). The ability to bind Ub permits Tsg101 to associate broadly with E3 ligases, although how it contributes to these diverse relationships is not clear. Tsg101 has been secondarily linked to the E3 enzyme, Mid1, during cytokinetic abscission [8] and to the E3 ligase Cbl, an enzyme involved in down-regulation of multiple receptor tyrosine kinases, through its interactions with Nedd4, a ligase that regulates the Cbl steady-state [9]. In the latter case, Tsg101 is recruited by Hrs, a protein that binds to the Cbl-modified receptors destined for degradation [10] and possesses a PSAP motif [11]. Tsg101 can also associate with E3 ligases Parkin and Mdm2 in autophagy [12].

The ability to employ the PT/SAP motif encoded in its structural precursor polyprotein, Gag, underlies the ability of HIV-1 and other viruses to effectively recruit Tsg101 to viral assembly sites [13,14,15,16]. The discovery of this exploitation elucidated the importance of the motif, but the Ub-binding properties of the protein are generally believed to merely aid PT/SAP-mediated recruitment. HIV-1 also encodes a LYPXXL motif that binds the V domain in the ESCRT adaptor Alix, a conduit to the E3 ligase Nedd4-1 [17]. Although Gag lacks a PPXY-type L domain that can bind E3 ligases directly, the cellular protein Angiomotin (AMOT) functions as an adaptor that binds both Nedd4L and HIV-1 Gag [18]. Interestingly, depletion of either ALIX or Tsg101 reduces virus release and infectivity, indicating that Nedd4-1 or Nedd4L, respectively, cannot substitute directly. Indeed, in the absence of direct Tsg101 binding to the PT/SAP motif in Gag, the Nedd4L-dependent rescue of virus production still requires the steady-state level of Tsg101 [19]. These proteins and their functional domains are summarized in Supplemental Figure S1.

We recently reported the use of paramagnetic relaxation enhancement (PRE) solution NMR to characterize complexes formed between the homologous to the E6AP carboxyl terminus (HECT) domain of a member of the neural precursor cell-expressed developmentally downregulated 4 (Nedd4) family of E3 Ub ligases and the UEV domain of Tsg101 [20]. The data revealed proximity between elements of the UEV domain involved in non-covalent Ub binding (α-hairpin and vestigial active sites) and sites on the front of the HECT domain (near the exosite) and back (near the αH1) [20]. Using an established reporter assay for the Nedd4-dependent rescue of HIV-1 budding [19,21] to quantify the impact of Ala substitution on HIV-1 particle production, we ascertained that the residues on the front face perturbed by UEV proximity defined determinants of infectious virus production [20]. Substitution of nearby residues not predicted to be in UEV proximity had little or no impact. Interestingly, however, while we observed 10- to 20-fold reductions in viral infectivity following mutation of these determinants, the impact on viral egress was significantly less (~2-fold). Since Mercenne et al. [18] had reported that AMOT depletion reduced the HIV-1 release and infectivity differentially (8-fold reduction in infectivity, 2.5-fold reduction in release) and especially when Tsg101 was depleted (20-fold reduction in infectivity, 3-fold reduction in virus release), we hypothesized that Nedd4L contributed to viral infectivity and budding by mechanisms likely mediated through distinct determinants. To test this notion, we substituted Ala for HECT residues in UEV proximity on the back face of the domain.

Here, we provide evidence that residues in UEV proximity housed on αH1, located on the back face of the HECT domain, define determinants of virus budding. The same mutations had no apparent effect in the context of a Nedd4-2s isomer whose N-terminal C2 domain is naturally truncated due to alternative splicing, precluding interactions between the N-terminal C2 domain and the C-terminal HECT domain responsible for stabilizing an enzymatically inactive conformation [22,23,24]. The results support the conclusion that the UEV–HECT interaction is a critical determinant of Nedd4 activation and control, therefore revealing a novel role for Tsg101 in virion morphogenesis. This function is distinct from the PT/SAP-binding-dependent recruitment of late-acting ESCRT membrane-scission machinery for virus particle release from infected cells. The results also suggest, unexpectedly, that UEV–HECT interaction drives completion of the immature Gag lattice and that interference with either the Tsg101 or Nedd4 function arrests particle assembly at an early stage.

## 2. Materials and Methods

### 2.1. NMR

Modeling of the interaction between Tsg101 UEV and Nedd4 HECT around the αH1 region used the top 10% of docked ensembles from our previously modeled interaction [20] involving restraints at Nedd4 sites 528 and 867 with the PDB ID: 5C7J orientation of Nedd4 HECT. For each UEV/HECT pair in these ensembles, contacts were defined as a residue in UEV coming within 4 Angstroms of a residue in HECT or vice versa, computed using python and Pymol. Contacts were summated across all ensemble members to generate the frequency histogram. 

### 2.2. Plasmids and Reagents

HIV-1 pNL4ΔEnv was as previously described with the p6 late domain altered by site-directed mutagenesis from the PTAP coding sequence to LIRL [20] (referred to as pNL4 LIRL). Nedd4L (Genbank, AAP75706.1) and Nedd4-2s (GenBank, AB007899.1) were gifts from F. Bouamr (NIAID). pLLEXP1-hTsg101-myc encoding full-length human Tsg101 NH2-terminally tagged with Myc was a gift from S. Cohen (Stanford University). Mutations in Nedd4L and Nedd4-2s were created using site-directed mutagenesis and confirmed by DNA sequencing. The numbering for amino acids targeted for mutagenesis in Nedd4-2s and Nedd4L is based on Nedd4L sequence (Genbank, AAP75706.1). For Western blots, primary antibodies were Rb anti-CA [25], mouse anti-actin (Sigma-Aldrich, St. Louis, MO, USA, A4700), mouse anti-Nedd4L (Santa Cruz Biotechnology, Santa Cruz, CA, USA, sc514954), mouse anti-Tsg101 (Santa Cruz Biotechnology, sc-7964), mouse anti Myc (Santa Cruz Biotechnology, sc-40). Secondary antibodies were goat anti-mouse IgG IRDye 680; goat anti-mouse IgG IRDye 800; and goat antirabbit IRDye800 (Li-Cor Biosciences, Lincoln, NE, USA). Benserazide (Cat# S2453) and tenatoprazole (Cat #S4212) were purchased from Selleck Chemicals LLC, Houston, TX, USA, solubilized in DMSO, and stored at −80 °C.

### 2.3. Virus Rescue Reporter Assay

293T cells (ATCC CRL-3216) were transfected using Roche X-tremeGene transfection reagent (Sigma-Aldrich). Briefly, at 24 h post transfection, cells were collected, washed in PBS, lysed (50 mM Tris, pH 7.4, 137 mM NaCl, 1.5 mM MgCl_2_, 1 mM EDTA, 1% Triton X-100, Roche cOmplete mini protease inhibitor cocktail) and centrifuged at 1000× *g* for 15 min. Supernatants were transferred to a clean tube, SDS PAGE sample buffer was added, followed by analysis by Western blotting. For analysis of the virus-like particles (VLPs), media from the cells were filtered (0.45 micron) and then pelleted through a 20% sucrose cushion by centrifuging at 20,000× *g* for 90 min. After centrifugation and washing, pellets were resuspended in SDS PAGE sample buffer/RIPA and examined by Western blotting. An infrared-based imaging system (Odyssey, LI-COR Biosciences) was used to measure the Western blot band intensities using Li-Cor Odyssey software, version 2.1.15. GraphPad Prism 9 software (GraphPad) was used to analyze the data.

### 2.4. Cell Proliferation (WST-1) Assay

293T cells were plated in 96-well tissue culture plates and allowed to grow for 24 h. Benserazide (K21) was added and after an additional 24 h, metabolic activity was analyzed using a cell proliferation reagent WST-1 (Sigma #5015944001) and measured on a colorimetric reader (Biorad iMark, Biorad Life Sciences, Hercules, CA, USA). The relative activity was plotted and calculated using GraphPad Prism 9 software.

### 2.5. ELISA, MAGI, and Electron Microscopy Analysis

For the measurement of specific infectivity, 293T cells were transfected and media collected at the times indicated in the Figure legends. The media was passed through an 0.45 micron filter, the media-associated p24 determined by an HIV-1 p24 Capture ELISA kit (ImmunoDX), and equal amounts of p24 used to infect Hela-CD4+-LTR-β-Gal cells. Infectivity was determined using the Multinuclear Activation of Galactosidase Indicator (MAGI) assay. For electron microscopy, 293T cells were grown on an ACLAR matrix and transfected. After 24 h, the cells were fixed in 2.5% EM-grade glutaraldehyde/PBS soaked in 2% osmium tetroxide followed by dehydration in a graded series of ethyl alcohol solutions and then embedded in durcupan resin. Thin sections of 80 nm were sliced and stained with uranyl acetate and lead citrate. Slices were viewed on a FEI Tecanai G2 Spirit BioTwin electron microscope.

## 3. Results

### 3.1. Residues Perturbed by UEV-HECT Proximity

**Figure 1:**
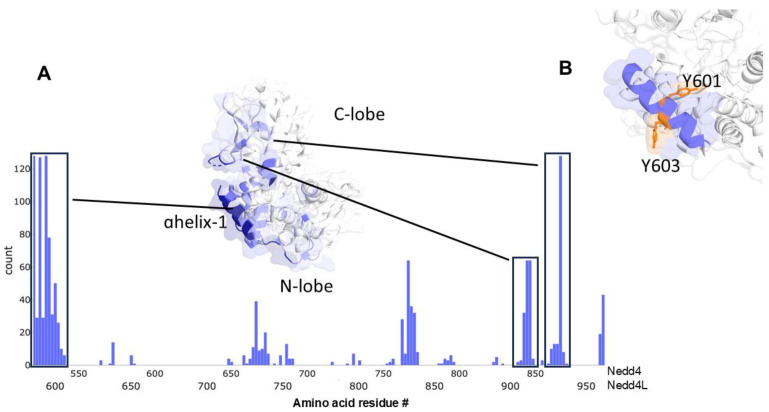
*(***A**) Frequencies of contacts observed between the Tsg101 UEV domain and the HECT domain of Nedd4. Contact frequencies are derived from the top 10% of ensemble pairs taken from modeling of solution NMR paramagnetic relaxation enhancement experiments [20] for labels at sites 528 and 867 in the Nedd4 HECT domain (PDB ID: 57CJ, ”L” orientation shown). Contacts were defined as a residue of Tsg101 UEV coming within 4 Angstroms of the corresponding HECT residue, where HECT residue numbers are given both for Nedd4 (top) and the equivalent position in Nedd4L (bottom). Two regions with the most contacts; αH1 and the region proximal to the catalytic cysteine in the C-lobe are highlighted. As Tsg101 UEV is structurally homologous to an E2 enzyme, interaction in this region far from the canonical E2 binding site was unexpected. (**B**) A view of the Nedd4L HECT domain taken from PDB ID: 3JVZ highlighting the region around αH-1 (blue). The determinants in this region (Y601 and Y603) investigated here are highlighted in orange.

Paramagnetic solution NMR characterization of a complex formed between the isolated HECT domain of the E3 ligase Nedd4-1 and the UEV domain of Tsg101 revealed that multiple sites on the HECT domain associated with elements of the Tsg101 UEV domain were involved in non-covalent Ub binding [20]; (Figure 1). Using an established assay [19,21] that reports Nedd4-dependent rescue of an HIV-1 mutant with impaired ability to recruit Tsg101 (NL4-LIRL), we found that HECT residues perturbed by UEV proximity defined determinants of viral egress and specific infectivity [20]. On the UEV side, the β-hairpin and the region proximal to the vestigial active site were in HECT proximity; both regions are sites of non-covalent Ub-binding [7,26]. On the HECT side αH1, the E2 binding site and the hinge connecting the N- and C-lobes of the HECT domain were in UEV proximity. Surface representation of αH1 and the residues (Y601 and Y603) on it predicted to be in greatest proximity is shown in Figure 1B.

### 3.2. UEV Proximity Impacts Enzyme Activity

**Figure 2:**
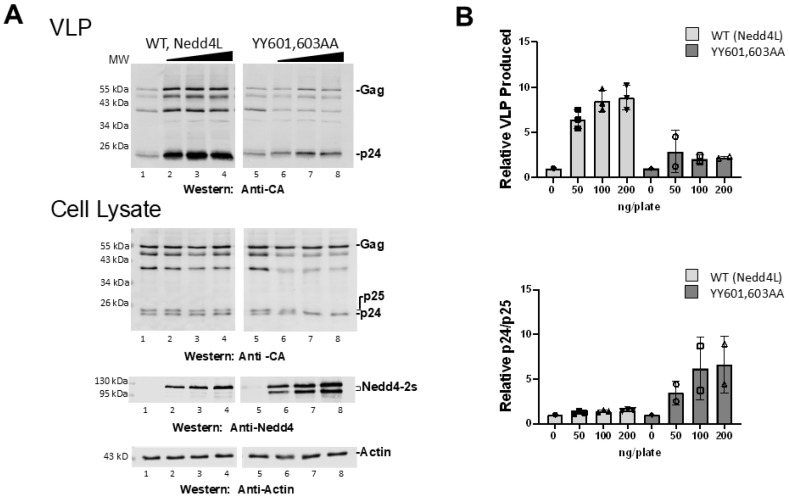
Mutation of Nedd4L HECT residues in UEV proximity prevented virus rescue. (**A**) 293T cells were transfected with pNL4-LIRL alone (lanes 1,5) or co-transfected with 50–200 ng Nedd4L WT (lanes 2–4) or Nedd4L YY601,603AA mutant (lanes 6–8). Top panel, levels of VLP detected by Western blots; bottom panel, Gag p24 and p25 in cell lysates. (**B**) Western blot signals were quantified relative to the pNL4-LIRL samples with no Nedd4. Top panel, VLP produced when co-transfected with Nedd4L WT or YY601,603AA mutant; bottom panel, the ratio of Gag p24 and p25 levels in cell lysates co-transfected with Nedd4L WT or YY601,603AA. The levels of VLP produced and the ratio of Gag p24 and p25 in the cell lysate were significantly different when co-transfected with the Nedd4L Y601/Y603AA mutant versus Nedd4L WT (Student’s *t*-test, *p* < 0.01). Number of independent trials, *n* = 2.

In our earlier study, mutations in the hinge region diminished HIV-1 specific infectivity to a significant extent but, surprisingly, had minimal effect on budding [20]. As the hinge determines relative N-lobe/C-lobe spatial orientation and therefore may reflect stages in Ub transfer between the HECT N-lobe regulatory and C-lobe catalytic regions, we hypothesized that residues located elsewhere in the domain regulated budding by fostering enzyme activation. Nedd4-1 and Nedd4-2/Nedd4L, but not the related Nedd4-2s isomer, are believed to be maintained in auto-inhibited states stabilized by interactions between their N-terminal C2 domain and their C-terminal WW4 or HECT (catalytic) domain [23,27,28]. The N-terminal region of Nedd4-2s is naturally truncated due to alternative splicing [29,30]. Our studies indicated that HECT αH1 (c.f., Figure 1B, blue) residues Y601 or Y603 (orange) were surface-accessible and in UEV proximity. We speculated that UEV proximity interfered with a C2-HECT interaction that stabilizes the autoinhibitory conformation. To test this notion, we determined the impact of Ala substitution of Y601,Y603. As shown in Figure 2A, VLP rescue promoted by WT Nedd4L (lanes 1–4) was significantly reduced (~5-fold) by Ala substitution of the UEV-proximal αH1 residues (lanes 5–8). Released VLP also exhibited defective CA maturation. These findings support the conclusion that UEV proximity defines HECT regions that impact the enzyme’s ability to rescue.

Interestingly, the inhibition resulting from the mutations was accompanied by significant stimulation of CA -SP1 proteolytic processing to mature CA in the cytoplasm, an event that is not promoted by the WT enzyme, as noted previously [19,21]. As defects in processing at the CA-SP1 junction accompany disruption in Tsg101 binding and consequent delays in HIV-1 budding [31], it was suggested that budding delays permit viral protease (PR), activated during egress, to reenter the cell and access Gag-related substrates in the cytoplasm. Finding that the αH1 mutations inhibited Nedd4L-mediated budding yet stimulated CA-SP1 processing in the cytoplasm (c.f., Figure 2B) suggested that interfering with Nedd4 activation altered accessibility of the CA-SP1 PR cleavage site. This implicates the ligase activity directly in the morphogenetic events that promote CA maturation. We noted that the CA-SP1 processing was accompanied by detection of an additional migrating form of Nedd4L that we presume to reflect a change in the relative levels of the Ub state of the protein [19,21,32]; however, we were unable to establish this directly.

### 3.3. C2 Domain Truncation Compensates for HECT αH1 Mutation

**Figure 3:**
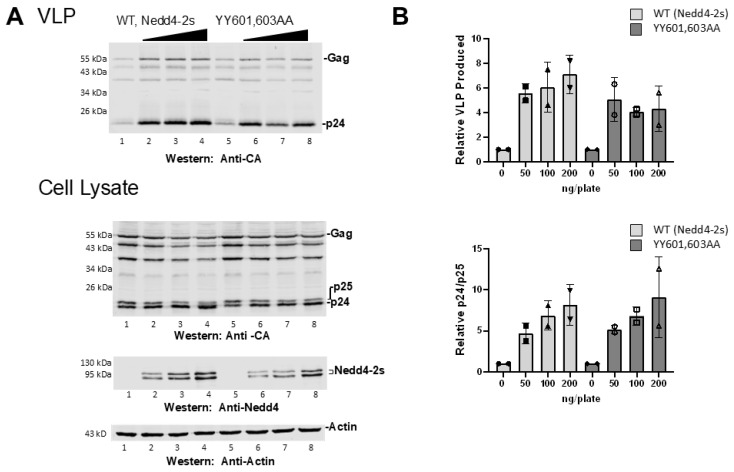
C2 domain truncation compensated for mutation of HECT residues in UEV proximity. (**A**) 293T cells were transfected with pNL4-LIRL alone (lanes 1,5) or co-transfected with 50–200 ng Nedd4-2s WT which has a naturally occurring C2 domain truncation relative to Nedd4L (lanes 2–4) or Nedd4-2s YY601,603AA mutant (lanes 6–8). Top panel, levels of VLP detected by Western blots; bottom panel, Gag p24 and p25 produced in cell lysates. (**B**) Western blot signals from rescue assays were quantified relative to the pNL4-LIRL samples with no Nedd4-2s assayed in parallel. Top panel, VLP produced when co-transfected with Nedd4-2s WT or YY601,603AA mutant; bottom panel, Gag p24/p25 ratios in cell lysates co-transfected with Nedd4-2s WT or YY601,603AA. The increase in VLP production and the ratio of p24/p25 for Nedd4-2s YY601,603AA were not significantly different from Nedd4-2s WT (Student’s *t*-test, *p* > 0.05). Number of independent trials, *n* = 2.

Previous studies identified residues in the C2 domain of some Nedd4-related enzymes that interact with their HECT domain, thereby supporting the notion that C2–HECT interaction maintains the autoinhibited state [23]. Independent studies reported that the Nedd4L/Nedd4-2 splice variant Nedd4-2s, whose C2 domain is naturally truncated, rescues HIV-1 release as efficiently as or more than the Nedd4L/Nedd4-2 isoform [19,21]. We therefore reasoned that, if UEV proximity interferes with C2-HECT autoinhibition, the impairment to Nedd4L rescue resulting from the Y601/Y603 mutations might be rescued by Nedd4-2s. Unlike Nedd4L/Nedd4-2, Nedd4-2s was observed to promote CA-SP1 processing [19,21]. As shown in Figure 3, the Nedd4-2s mutant possessing the Y601/Y603A substitution was as competent for virus rescue as its parent (Figure 3A, compare lane 1 to lanes 2–4 and lane 5 to lanes 6–8, summarized in Figure 3B), supporting the prediction. As expected, WT Nedd4-2s stimulated CA-SP1 processing and the mutations had no detectable effect (Figure 3B). Both WT and mutated Nedd4-2s exhibited more than one form of the enzyme by SDS-PAGE, indicating that the presumed Ub modification state of the protein was maintained in the Nedd4-2s context irrespective of the αH1 mutations. We conclude that the C2 domain truncation relieved the inhibitory effect of the HECT αH1 mutations on virus egress without apparent effect on other aspects of the parental enzyme activity. These findings support the hypothesis that UEV proximity disrupted C2-HECT autoinhibition mediated or regulated by Y601/Y603 in αH1.

### 3.4. Deletion of the Entire αH1 Abrogates Virus Rescue and CA-SP1 Processing

**Figure 4:**
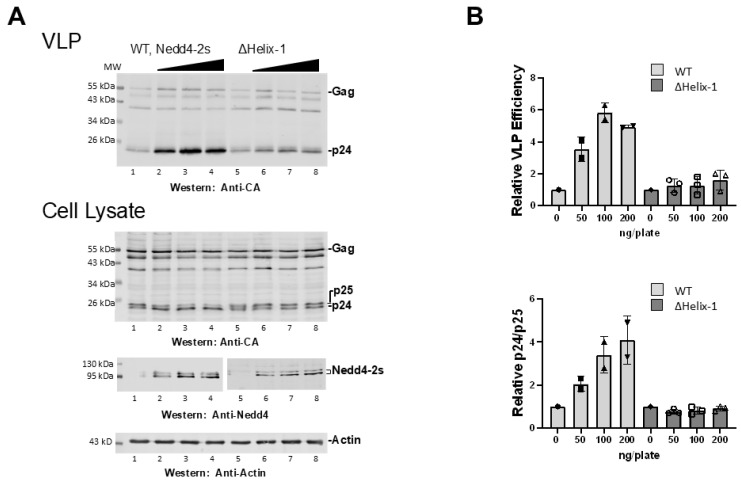
Deletion of the HECT domain αH1 abrogates virus rescue. (**A**) 293T cells were transfected with pNL4-3-LIRL alone (lanes 1,5) or co-transfected with 50–200 ng Nedd4-2s WT (lanes 2–4) or Nedd4-2s-ΔαH1 (lanes 6–8). The Nedd4-2s constructs were compared for their ability to rescue pNL4-LIRL budding as measured by the levels of VLP detected by Western blots (top panel) and Gag p24 and p25 in cell lysates (bottom panel). (**B**) Western blot signals from rescue assays were quantified relative to the pNL4-LIRL samples with no Nedd4-2s assayed in parallel. Top panel, VLP produced when co-transfected with Nedd4-2s WT or Nedd4-2s-ΔαH1; bottom panel, the ratio of Gag p24 and p25 for Nedd4-2s WT or Nedd4-2s-ΔαH1. The VLP efficiency and the ratio of p24/p25 for ΔαH1 mutant were significantly different from WT (Student’s *t*-test, *p* < 0.01). Number of independent trials, *n* = 2.

Early structural studies did not include αH1 in engineered constructs and only relatively recently has it been identified as a conserved element involved in regulation of HECT domain auto-ubiquitylation [33]. Indeed, earlier studies demonstrated that chimeric Nedd4-2s enzymes lacking αH1 and a proximal region (aa 593–639) can rescue Leu-zipper modified Gag proteins [32]. As αH1 (aa~593–604) is a major site of UEV interaction [20]; (c.f., Figure 1), we determined the effect of its deletion on Nedd4-2s-mediated virus rescue. As shown in Figure 4, deletion of αH1 (R_595_EFKQKYDYF_604_) from Nedd4-2s reduced VLP rescue significantly. Stimulation of CA-SP1 processing was also abrogated. The results indicate that the element by itself is an important contributor to both virus rescue and temporal regulation of CA maturation, an event that normally follows egress. These observations support the conclusion that its UEV proximity serves to modulate the activity of the enzyme.

### 3.5. Tsg101_UEV-HECT_Nedd4L Interaction Promotes Gag Lattice Completion

**Figure 5:**
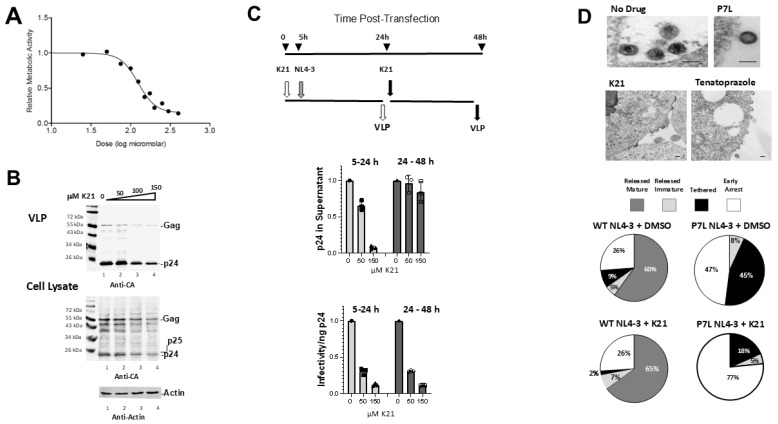
Benserazide (K21) arrests budding of pNL4-3-P7L. (**A**) WST-1 assay of cell metabolic activity at increasing concentrations of K21 (CC50 110–140 µM, 95% CI); (**B**) Effect of K21 (50 µM) on VLP production. Top panel, Western analysis of virus-like particles (VLPs); bottom panel, Gag proteins in cell lysate for 293T cells transfected with pNL4-3 WT. (**C**) Effect of early versus late K21 (50 µM) addition on VLP production as determined by ELISA and MAGI assays (*n* = 2). Numbers were normalized to the untreated (DMSO) controls. (**D**) Examination by electron microscopy of mock, K21-treated cells, and Tenatoprazole-treated cells transfected with NL4-3-P7L (top). Bottom, Quantitative analysis of budding morphologies in cells exposed to either DMSO or 50 µM K21. For P7L, distribution of morphologies for control (DMSO) versus K21 were significantly different (Chi square test, *p* < 0.001); for WT, distribution was not significantly affected by K21.

We reported earlier [34] that prazoles, which interfere with Tsg101 UEV Ub-binding, arrest budding at a stage prior to formation of the canonical “lollipops” that accumulate following depletion of Tsg101 [14]. We therefore hypothesized that interference with the functioning of Nedd4L might result in a similar defect, as suggested by the findings of Mercenne et al., where depletion of the endogenous pool of AMOT arrested budding at an early stage [18]. To block Nedd4 binding, we employed the small molecule Benserazide (designated as K21; [35]). Benserazide, a well-tolerated prodrug used in combination with Levodopa to treat Parkinson’s disease, was identified in a screen for small molecules capable of binding to the Nedd4 family members WWP1 and WWP2 and interfering with the binding of PY-containing peptides. As shown in Figure 5, the drug exhibited low cytotoxicity in HeLa and 293T cells with cytotoxic concentration (CC50) values of 110–140 μM (95% confidence interval) (Figure 5A) and moderate inhibitory activity against pNL4-3-WT as judged by Western blots (Figure 5B). In a single round replication assay, the drug was most effective in arresting budding when added 5–6 h prior to pNL4-3-WT transfection, as is the case for prazole-mediated inhibition [34,36]. However, it was effective at impairing production of the infectious virus when added early or late (Figure 5C). No significant differences in early or late structures were detected in the untreated samples versus treated samples for cells transfected with pNL4-3 WT. However, early arrest similar to that induced by AMOT depletion [18] was apparent when comparing untreated versus treated samples if cells were transfected with pNL4-3-P7L (Figure 5D, top), where Tsg101 binding is impaired and budding is Alix/Nedd4-1-dependent [17] (Figure 5D, bottom). This finding supports the conclusion that the block results from interference with Tsg101–Nedd4 interaction. The drug was also effective in blocking virus spread in Jurkat cells (Supplemental Figure S2).

## 4. Discussion

Downregulation of Ub ligase activity prevents premature or inappropriate ubiquitination and is critical for maintaining cellular homeostasis. The enzymes are believed to adopt an autoinhibited state in which oligomerization or interactions with elements upstream of the catalytic HECT domain, i.e., the C2 and/or WW domains, sequester the HECT domain in a multi-lock mechanism. Family members WWP1, WWP2, and Itch utilize a highly similar multi-lock autoinhibition mechanism where ligase regulation is driven mainly by multiple WW regions, whereas the C2 domains in Nedd4-1, Nedd4 L (Nedd4-2), and Smurf play a key regulatory role [22] with a variable contribution from the WW domain [27]. Our studies indicate that the Ub-binding regions on the UEV domain of Tsg101 that lie in proximity to the αH1 on the HECT domain of Nedd4 are determinants of ligase activation. Interference with Nedd4 substrate recognition or with Tsg101 UEV Ub-binding arrested budding at an early stage, i.e., prior to the obvious bud neck formation that reflects delayed recruitment of ESCRT-III. Both Nedd4-mediated rescue and ESCRT-III recruitment require events mediated through the UEV domain in Tsg101 [14,19] that house Ub- and PT/SAP-binding pockets. Therefore, these observations collectively imply that two distinct events, Gag morphogenesis and VLP budding, are required for complete Gag assembly and that Tsg101 engagement of Nedd4 may take place prior to direct engagement of PT/SAP by Gag. It is noteworthy that Hrs, a component of ESCRT-0 whose actions are mimicked by HIV-1 Gag [37], engages Nedd4 for cargo capture prior to interacting via its PSAP motif with Tsg101/ESCRT-I [11,38,39].

As suggested by the results of previous studies [14,18,40,41], where diverse factors were found to spatially and/or temporally influence events in Gag assembly and egress, both events, i.e., particle morphogenesis and VLP budding, can be kinetic bottlenecks. It is now well established that actions that interfere with recruitment of ESCRT-III result in accumulation of “lollipop” structures (i.e., buds with extended necks). Our results, demonstrating that interference with Nedd4L C2–HECT domain interaction at regions in UEV proximity prevented VLP release but was rescued by substituting constitutively active Nedd4-2s, directly implicate Tsg101 in Nedd4 activation. Ku et al. [40] proposed that shortcomings in Gag interactions with essential cellular components, might be responsible for “pauses” in Gag assembly at a stage prior to induction of the membrane curvature. They specifically suggested Ub ligases, enzymes shown to play a role in HIV-1 budding [17,19,20,21,42]. Supporting their suggestion, siRNA-mediated knockdown of adaptor proteins such as AMOT also reduce viral particle production and arrest viral bud formation at a half-completion stage [18,41] very similar in appearance to our findings employing small molecules that disrupt Tsg101-Ub binding or Nedd4 function ([34]; c.f., Figure 5). Their hypothesis, that Ub ligases play critical roles, is directly supported by the findings described here that Tsg101–Nedd4 interaction regulates viral budding.

As noted above, early structural studies did not include αH1 in engineered constructs. Using in silico bioinformatic analyses coupled with secondary structural prediction software, Kane et al. provided evidence that the N-terminal αH1 of the HECT domain, predicted in all 28 human HECT E3 ubiquitin ligases, forms an obligate amphipathic α-helix that binds to a hydrophobic pocket within the HECT N-terminal lobe. UEV-mediated activation through αH1 would be thus highly conserved. In the case of HECT family members, Nedd4L and SMURF, residues in this region were reported to communicate through conformational changes with αH1, which is located on the opposite face of the domain [22,23,27,43]. Also interestingly, in previous structural studies, αH1 has been suggested to stabilize the compact “T-shaped” structure in the related HUWE1 and WWP1 HECT E3 enzymes. Possibly then, UEV proximity to αH1 influences N- and C-lobe dynamics which, in turn, are expected to influence Ub acquisition. Collectively, these observations suggest that structural “cross-talk” between HECT and UEV proximal regions is plausible as a mechanism of Ub signaling for several of the HECT type enzymes.

**Figure 6:**
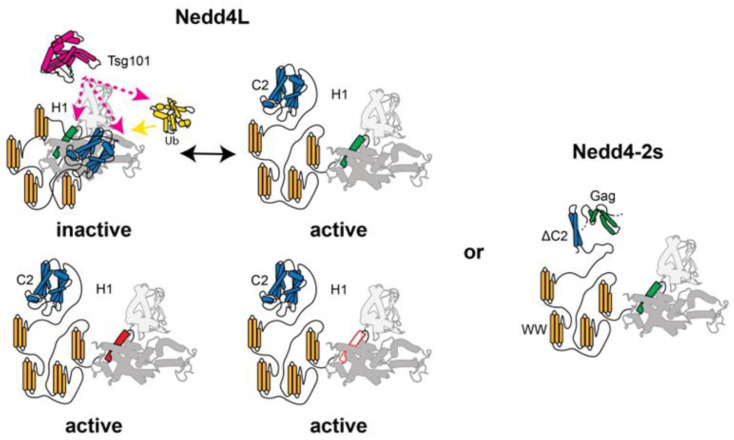
Model: Tsg101 UEV interference with Nedd4 autoinhibition. Nedd4L is normally in an autoinhibited, inactive state (top left) induced by backbinding of the N-terminal C2 domain (blue) and WW domains (orange) to the catalytic HECT domain (gray). This form exists alongside the active form (top right), which may be induced by interaction of the Tsg101 UEV domain (pink), which in turn may interact with Ub (yellow) on the HECT domain or through direct interaction with the HECT domain or αH1 region (green). Amino acid substitutions in αH1 (bottom left) presumptively alleviates the autoinhibited state and Tsg101 interaction, similarly to the deletion of the entire αH1 region (bottom right). Mutation or deletion of αH1 has no impact on the Nedd4-2s isoform (right panel), whose truncated C2 domain putatively prevents backbinding and formation of the autoinhibited state. The Nedd4-2s isoform is distinct from the FL in its ability to interact with HIV-1 Gag, whose binding potentially affects helix-1 (far right, green).

The model in Figure 6 summarizes how Tsg101 can activate HECT through different interaction schemes. A direct interaction with HECT can displace the C2 domain of Nedd4 from back-binding to and inhibiting HECT. Similarly, Tsg101 interaction around the α1 helix also induces a conformation of this helix that favors the active form of the enzyme. Finally, an interaction between Tsg101 and Ub on the HECT seems to also contribute to the activation of the enzyme. These events may occur sequentially to enhance the activation of HECT, or they may act independently to provide different pathways for HECT activation. There is one tantalizing general feature: Tsg101 serves as a ubiquitin receptor that recognizes proteins that become ubiquitinated, such as HIV-1 Gag, Tsg101/ESCRT-I itself and/or Nedd4 through the ability of its UEV domain to bind Ub non-covalently. The general functional consequence of Tsg101 as a mimic of E2 is an open question that we hope to address in the future.

In summary, here we provided evidence that the upstream ESCRT-I factor Tsg101 plays a crucial early role in Gag assembly and budding through its ability to activate the E3 ligase Nedd4 and promote clustering of Gag assemblages that is sufficient to stimulate CA-SP1 processing, a critical activating event in infectious HIV-1 particle morphogenesis. Our studies indicate that Ub, long implicated as a critical factor, and Tsg101-Nedd4 interaction play crucial initial roles distinct from mere support of PT/SAP-mediated Tsg101 recruitment. Our studies reveal a previously uncharacterized dynamic relationship between the E3 Ub ligase Nedd4 and the Ub E2 variant Tsg101 protein that suggests that tight control of the ligase activity is a previously unappreciated function of Tsg101. This newly revealed activity expands the number of ways that Tsg101 function could be targeted for anti-viral drug development, as demonstrated here by effective small molecule interference.

## Data Availability

All data presented in this study are summarized in the paper. The detailed data of this study are available on request from the corresponding author.

## Supplementary Materials

The following supporting information can be downloaded at: https://www.mdpi.com/article/10.3390/v16101566/s1, Figure S1: Retroviral L domains are conduits to E3 ligases; Figure S2: Benserazide (K21) inhibition of HIV-1 transmission in a spreading infection in Jurkat cells. Reference [44] was cited in the Supplementary Materials.
