# Supplementary material for: Tsg101 UEV Interaction with Nedd4 HECT Relieves E3 Ligase Auto-Inhibition, Promoting HIV-1 Assembly and CA-SP1 Maturation Cleavage
Source: Viruses. 2024 Oct 2;16(10):1566. doi: 10.3390/v16101566 (PMC11512315; doi:10.3390/v16101566)
Supplement: Supplementary file 1 [file viruses-16-01566-s001.zip › viruses-3207814-supplementary.pdf]

viruses MDPI

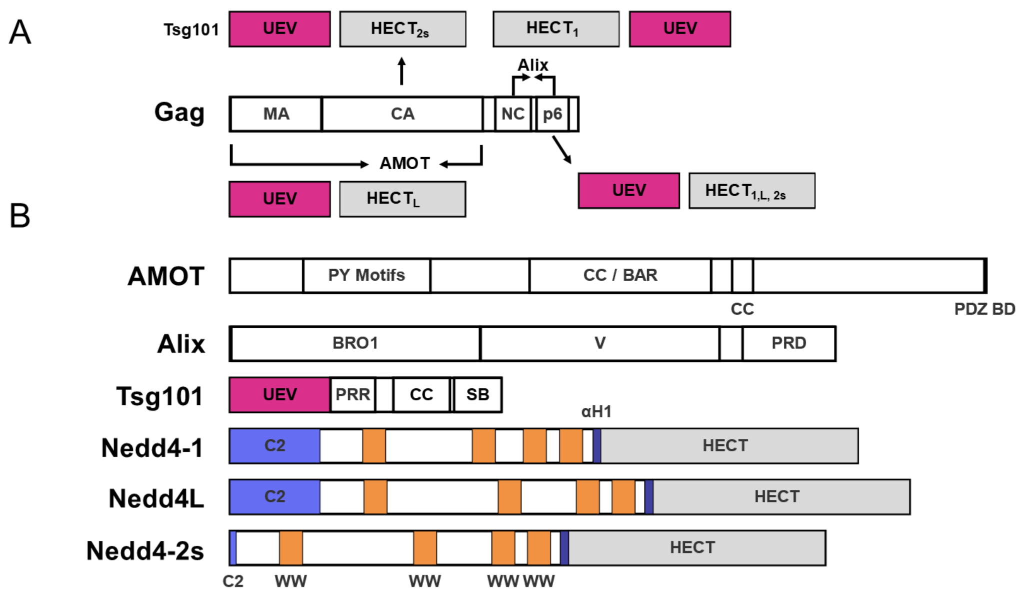


**Figure S1.** Retroviral L domains are conduits to E3 ligases. (**A**) Nyenhuis et al. 2023 reported that Tsg101 (UEV) and Nedd4 (HECT) are in proximity in solution and confirmed the findings of others [17,19,21,32] that the HECT domains of several Nedd4 family members can rescue Gag unable to recruit Tsg101 directly, i.e., the L domains of retroviruses (and other viruses) are conduits to E3 ligases. Accessing Nedd4, however, still required the presence of Tsg101 [19]. This is likely what makes the other L domain pathways less efficient than PT/SAP. (**B**) Schematic of proteins involved in this study. Alix and Angiomotin are adaptor proteins that bind to HIV-1 Gag and link it to Nedd4 family members Nedd4-1 and Nedd4L, respectively [17,18]. Tsg101 has an N-terminal UEV domain (magenta), which is homologous to E2 enzymes, and C-terminal coiled-coil and steadiness-box regions, which are involved in ESCRT complexation. Nedd4 family members typically have an N-terminal C2 domain (blue) followed by several substrate-recognition WW domains (orange), and the catalytic HECT domain (gray). The HECT domain consists of N- and C-lobes connected by a flexible hinge and flanked by an N-terminal alpha helix, αH1. Domains are annotated for Angiomotin (AMOT, Uniprot Q4VCS5-1), the ESCRT adaptor Alix (Uniprot Q8WUM4-1), the ESCRT-I protein Tsg101 (Uniprot Q99816) and the HECT E3 ligases Nedd4 (Uniprot P46934-4), Nedd4L (Uniprot Q96PU5-1), and its variant with truncated C2 domain Nedd4-2s (Uniprot Q96PU5-4). Graphics were made using IBS2.0 [*]. Proteins are drawn to scale except for the HECT domains in Panel A. *IBS 2.0: an upgraded illustrator for the visualization of biological sequences.[44}

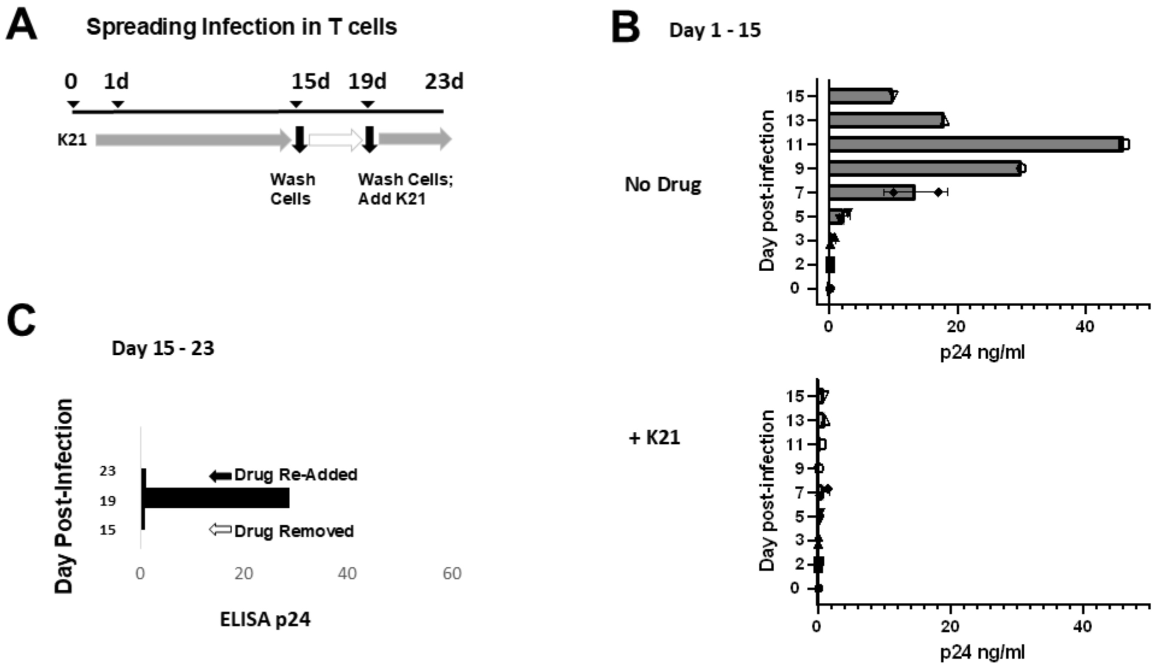


**Figure S2.** Benserazide (K21) inhibition of HIV-1 transmission in a spreading infection in Jurkat cells. (**A**), Schematic diagram summarizing experimental protocol. Jurkat cells (triplicate samples of $5 \times 10^5$/well) were incubated for 2 h with HIV-1 NL4-3 in treatment media containing 50 µM Benserazide (K21) or a control (DMSO). At the end of this period, unbound virus was removed by centrifugation and the cells washed once, resuspended in fresh media and returned to the 37 °C incubator. For the next 15 days, tissue culture media was removed daily by centrifugation and saved for virus measurement by p24 capture ELISA and cells were replenished with fresh control or K21 treatment media. (**B**) ELISA readings, Days 1–15. In control media (containing DMSO), virus replication peaked at day 11; in the presence of K21, production peaked at 13 days. A comparison of peak values indicated that K21 reduced virus production ~10-fold. Cells were monitored periodically for viability by Trypan Blue assay. (**C**) ELISA readings, Days 15–23. To test their ability to produce virus after sustained exposure, at day 15 the K21-treated cells were washed, fresh media without inhibitor was added and the cells were incubated 4 days longer (to day 19). The supernatant was collected, the cells were washed again and then incubated for another 4 days in media with inhibitor, adding fresh inhibitor daily. The final supernatant was collected on day 23. The virus level surged by 15-fold when K21 was removed indicating that the observed inhibition was not attributed to irreversible cell toxicity. It then dropped by 15-fold upon re-addition of K21, indicating that the virus was still susceptible. Thus, the cells maintained the ability to produce virus and the virus in the population at 15 days was essentially as susceptible to K21 as the initial virus population used to infect the Jurkat cells. Number of independent trials: $n = 2$.
